# Characterization of the transient middle cerebral artery occlusion model of ischemic stroke in a HuR transgenic mouse line

**DOI:** 10.1016/j.dib.2017.10.033

**Published:** 2017-10-17

**Authors:** A. Ardelt, R. Carpenter, I. Iwuchukwu, A. Zhang, W. Lin, E. Kosciuczuk, C. Hinkson, T. Rebeiz, S. Reitz, P. King

**Affiliations:** a Department of Neurology, University of Chicago, 5841 S. Maryland Ave, MC2030, Chicago, IL 60637, USA; b Department of Neurocritical Care, Ochsner Medical Center, 1514 Jefferson Hwy., New Orleans, LA 70121, USA; c Division of Hematology-Oncology, Northwestern University, 675 North St. Clair, Chicago, IL 60611, USA; d Department of Neurology, University of Alabama at Birmingham, 1720 7th Sve South and the Birmingham VA Medical Center, Birmingham, AL 35233, USA

## Abstract

This set of experiments characterizes a model of transient cerebral ischemic stroke in a transgenic (Tg) mouse line in which the glial fibrillary acidic protein (GFAP) promoter is utilized to drive expression of a human RNA-binding protein, HuR. Additionally, the effect of cerebral ischemia on the expression of endogenous Hu proteins is presented.

**Specifications Table**

**Table:** 

Subject area	*Biology*
More specific subject area	*Animal models of human disease*
Type of data	*Text, tables, and figures*
How data was acquired	*Microscope; physiologic monitors*
Data format	*Analyzed*
Experimental factors	*Wild-type versus genetically modified mice; transient cerebral ischemia*
Experimental features	*The transient middle cerebral artery occlusion model was tested in a new genetically modified mouse line ectopically expressing the RNA-binding protein, HuR, and the effects of transient ischemia on endogenous RNA-binding proteins including Hu proteins were investigated in wild-type mice as well as rats*
Data source location	*Chicago, IL and Birmingham, AL, USA*
Data accessibility	*Data is located in this article*

**Value of the data**

• The HuR transgenic line is suitable for further investigations of cerebral vascular physiology and pathology
• The differential effects of ischemia on endogenous RNA-binding proteins including Hu proteins suggest further studies on their response to ischemia and therapeutic potential.

## 1 Data

Cerebral vascular anatomy, physiologic parameters, and infarct histology are shown in HuR transgenic (Tg) mice compared to wild-type littermates with and without transient cerebral ischemia. Additionally, the effects of transient cerebral ischemia on endogenous RNA-binding proteins including Hu proteins are shown.

### 1.1 Characterization of the ischemic stroke model in transgenic (Tg) mice

Although there was circle of Willis variability from animal to animal, there were no transgene-associated differences (Table 1). There were no transgene-related differences in normal (non-ischemic) physiology (data not shown). In ischemic physiology controls, there was a difference in P_a_O_2_ between the groups, but biologically insignificant since P_a_O_2_ was >100 mm Hg in both (Table 2). In the 72-hour tMCAO cohorts, a 0.1-degree rectal temperature difference was not transgene-related (Table 3). There were three deaths in the 72-hour survival group: one Wt animal and two Tg animals. One Tg animal in the 72-hour survival group was excluded due to hemorrhagic conversion of the infarct.

**Table 1:** Posterior communicating artery (PCOM) configuration.

	Wt, n = 8	Tg, n = 9
Bilateral PCOMs	2	4
Right PCOM only	4	4
Left PCOM only	1	1
No PCOMs	1	0

Non-significant.

**Table 2:** Ischemic parameters in the ischemic physiology cohort.

	Wt, n = 6	Tg, n = 6
Weight, g	26 ± 4	28 ± 1
Occlusion, %	11 ± 4	11 (10-12)
MAP, mmHg	85 ± 6	85 ± 2
Temperature, ^o^C	36.8 ± 0.2	36.8 ± 0.1
pH	7.35 ± 0.04	7.34 ± 0.05
p_a_CO_2_, mmHg	35 ± 8	40 ± 7
p_a_O_2_, mmHg	153 ± 26*	114 ± 15*
Hematocrit, %	38 ± 5	42 ± 2
Glucose, mg/dL	177 ± 16	210 ± 58

Mean +/- SD or median (IQR). Non-significant except * *t*-test, p=0.011.

**Table 3:** Ischemic parameters in transient middle cerebral artery occlusion (tMCAO) experimental cohorts.

	24 h Survival		72 h Survival	
	Wt, n = 20	Tg, n = 20	Wt, n = 26	Tg, n = 23
Weight, g	23 (22-25)	24 ± 3	24 ± 3	23 ± 3
Occlusion, % baseline	10 ± 4	10 ± 3	9 (7-10)	11 ± 6
Temperature, ^o^C	36.8 ± 0.2	36.9 ± 0.2	36.8 (36.7-36.9)*	36.9 (36.8-37.0)*
Reperfusion, % baseline	47 ± 18	54 ± 24	48 ± 25	41 ± 16

Mean +/- SD or median (IQR). Non-significant except * Mann-Whitney rank sum test, p = 0.004.

### 1.2 Infarct histology in Tg and Wt mice

**Fig. 2:**
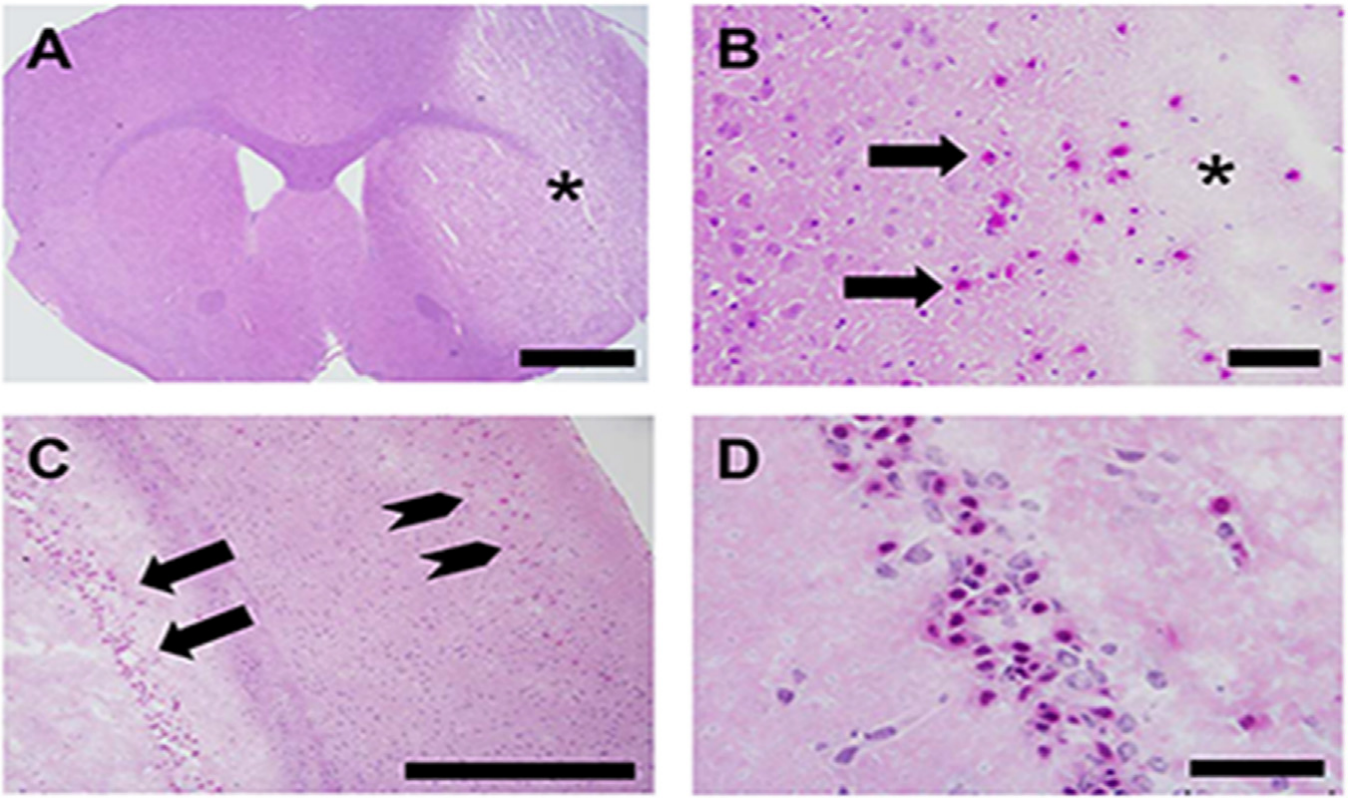
Histology in Tg mice. (A) Neuropil rarefaction (asterisk). 1.25x; scale bar = 1 cm. (B) Red neurons (arrows) and neuropil vacuolation (asterisk). 20x; scale bar = 50 μm. (C) Hippocampal (arrows) and cortical (arrowheads) infarcts. 10x; scale bar = 500 μm. (D) High-power image of hippocampal injury. 60x; scale bar = 50 μm. (For interpretation of the references to color in this figure legend, the reader is referred to the web version of this article.).

Striatal and cortical infarcts in both cohorts were characterized by pyknotic nuclei, cytoplasmic eosinophilia and shrinkage, enlarged pericellular spaces and vacuolation of the neuropil (Fig. 2 A, B). In some Tg and Wt animals, infarcts involved regions beyond the striatum and cortex (Fig. 2 C, D; Table 4). Such infarcts were represented equally in the cohorts: 24h, 4/8 (Wt) vs. 6/11 (Tg), Fisher’s exact test, non-significant; 72 h, 8/10 (Wt) vs. 7/10 (Tg), Fisher’s exact test, non-significant.

**Table 4:** Regional distribution of ischemic injury.

	Wt, 24 h (n = 8)	Tg, 24 h (n = 11)	Wt, 72 h (n = 10)	Tg, 72 h (n = 10)
Striatum	8	11	10	10
Rostral^a^ cortex	8	11	10	10
Caudal^b^ cortex	7^c^	11	9	10
Hippocampus	4	6	7	7
Amygdala	2	2	5	4
Thalamus	3	3	3	2
Hypothalamus	1	1	0	0
Midbrain	1	3	3	2

Non-significant.

a Bregma 1.10 to -2.54.

b bregma -3.64.

c the most caudal. Tissue section from one animal was lost, i.e., n = 7.

### 1.3 Ischemia-reperfusion affects endogenous RBPs and Hu proteins in Wt mice

**Fig. 1:**
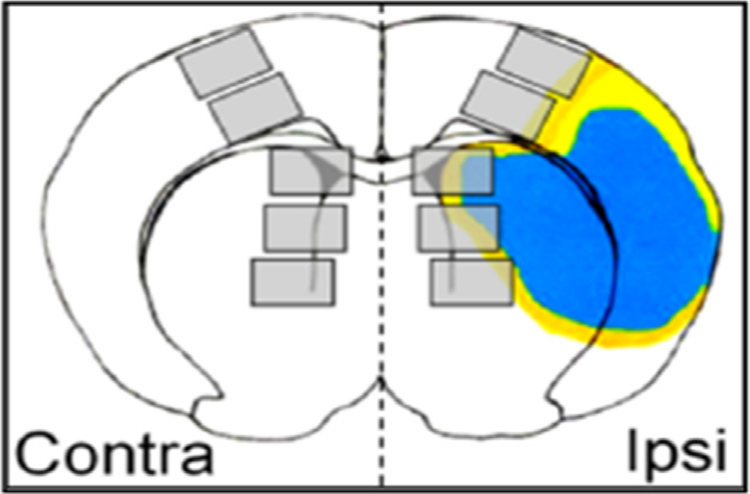
Reference diagram: infarct core (blue) and borderzone (yellow) and approximate locations of ipsilateral (Ipsi) and contralateral (Contra) ROI (grey boxes), not to scale. (For interpretation of the references to color in this figure legend, the reader is referred to the web version of this article.).

**Fig. 3:**
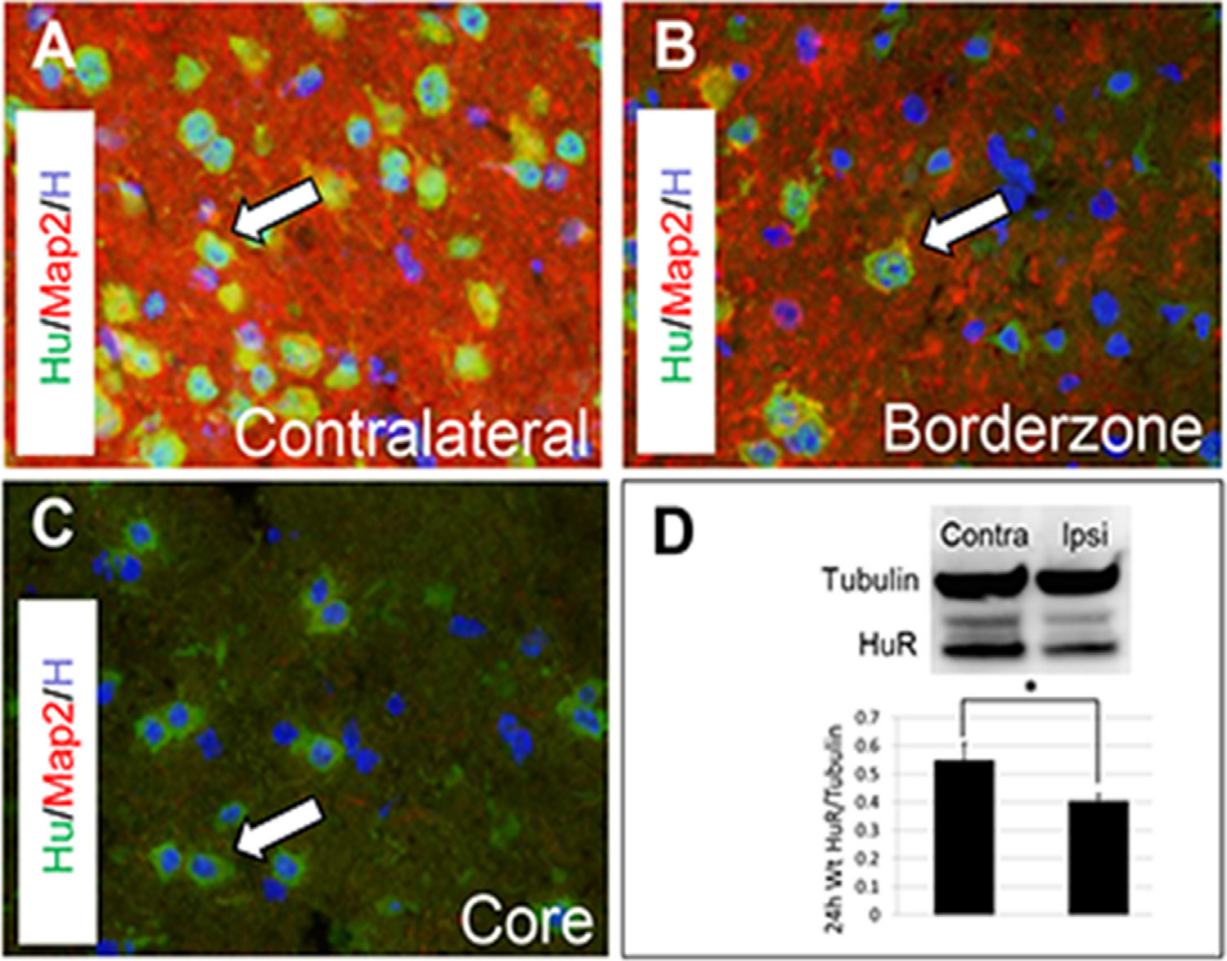
Distribution of endogenous Hu proteins in Wt mice 24 hours after injury. (A) Hu immunoreactivity (green) with nuclear and cytoplasmic distribution (arrow) in contralateral ROIs. (B) Decrease in Hu immunoreactivity (arrow) and Map2 immunofluorescence in the infarct borderzone. (C) Lack of Map2 signal and predominantly cytoplasmic Hu immunoreactivity (arrow) in the infarct core. (D) HuR/tubulin Western blot with densitometry, HuR normalized to tubulin, n = 6, mean ± SEM, *p = 0.046 (Student’s t-test). (For interpretation of the references to color in this figure legend, the reader is referred to the web version of this article.)

Using an antibody which immunostains HuR and the neuron-specific members of the Hu family, abundant immunoreactivity in contralateral ROIs was demonstrated (Fig. 1, Fig. 3 A). Hu immunoreactivity was attenuated in the ischemic lesion (borderzone and core) (Fig. 3 B, C). Western blotting confirmed a decrease of HuR by ~ 30% in the ipsilateral hemisphere 24 hours after tMCAO (Fig. 3 D). In the ischemic core, Hu immunoreactivity was observed in the cytoplasm of remaining cells, whereas in contralateral tissue it was present primarily in nuclei (Fig. 3 A, C).

### 1.4 Ischemia-reperfusion affects endogenous RBPs including Hu proteins in rats

**Fig. 4:**
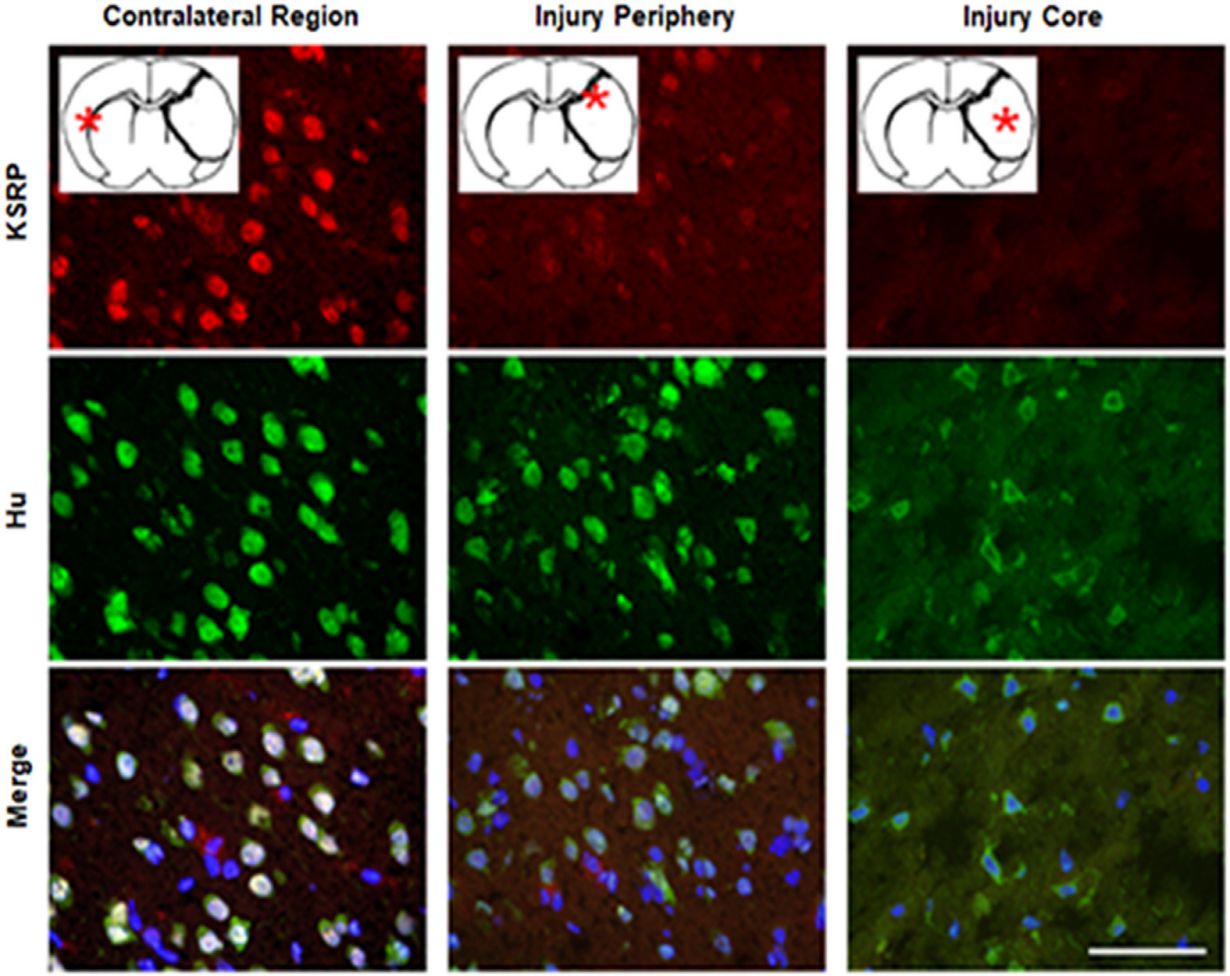
The effect of ischemia-reperfusion on Hu immunoreactivity in rats. Approximate locations of the images are demonstrated with asterisks in the diagrams: the stippled border represents the infarct borderzone. Top row: KSRP immunoreactivity in uninjured contralateral region (left column); periphery of injury, i.e., the region immediately adjacent to where KSRP immunoreactivity transitions to the altered pattern (middle column); and core of injury (right column). Middle row: Hu immunoreactivity in the same regions. Bottom row: merge of KSRP and Hu immunoreactivity, and Hoechst nuclear stain. In the contralateral region, there is colocalization of nuclear KSRP and Hu immunoreactivity in nuclei of most cells. Hu immunoreactivity is also cytoplasmic in some of the cells. On the ipsilateral side, at the periphery of injury, KSRP immunoreactivity attenuation appears greater than Hu immunoreactivity attenuation. In the core of injury, KSRP immunoreactivity is absent, and Hu immunoreactivity is located in the cytoplasm and processes of shrunken cells with preserved nuclei. Scale bar = 100 μm. (For interpretation of the references to color in this figure legend, the reader is referred to the web version of this article.).

In a similar ischemic stroke model in rats, we observed a comparable localization of Hu immunoreactivity (Fig. 4, middle row). Unlike Hu proteins, KSRP was attenuated at the periphery of the lesion and was undetectable in the core (Fig. 4, top row).

## 2 Experimental design, materials and methods

### 2.1 Animals

Procedures were approved by the University of Chicago Institutional Animal Care and Use Committee and were in accordance with NIH guidelines for the use of animals in research. The Tg mouse line was previously described [4]: the GFAP promoter was utilized to direct expression of the human HuR gene coupled with the FLAG octapeptide tag in C57Bl/6 J mice. Animals were housed in cages with littermates on a 6 a.m. - 6 p.m. light cycle. Mice were fed Teklad Diet 2918 (Harlan Laboratories, Madison, WI) *ad libitum*. Technicians and investigators were blinded to the genetics during performance of the experiments. Wild-type (Wt) and Tg mice are externally indistinguishable. Mice were randomly tagged with sequentially numbered ear tags at weaning and underwent experimental ischemic stroke when 8–10 weeks old. Male mice were utilized for the experiments. Mice were randomly selected from the cages for the following control experiments: circle of Willis assessment; normal physiology; ischemic physiology; and histology. Wt mice and rats were used in evaluating the expression of endogenous RNA-binding proteins in normal and ischemic brain.

### 2.2 Circle of Willis

Control mice were terminally anesthetized with 5% isoflurane in oxygen (0.05 L/min) and medical air (1 L/min), and 5 ml of 37 °C phosphate-buffered saline (PBS) followed by 5 ml of 75% acrylic latex (Premium Plus Pure Black #8620, Behr Process Corp., Santa Ana, CA) in PBS were injected via cardiac puncture. Brains were removed, fixed in 10% buffered formalin (Fisher Scientific, Fair Lawn, NJ), and visualized with a surgical microscope (Olympus SZX7, Olympus America Inc., Lombard, IL).

### 2.3 Transient middle cerebral artery occlusion (tMCAO)

Mice were anesthetized with isoflurane (induction: 5%; maintenance: 1.5–2%) in oxygen (0.05 L/min) and medical air (1 L/min). After sterile preparation and draping, tMCAO was performed as previously described [1] with minor modifications. Briefly, the right common carotid artery (CCA) was exposed, and a 7-0 nylon suture tipped with dental adhesive was introduced into the CCA until laser Doppler signal (Periflux System 5000, Perimed, Inc., Ardmore, PA) from a probe positioned over the MCA territory dropped by ≥80%. During MCAO, rectal temperature was maintained between 36.5 and 37.5 °C with a homeothermic system (Harvard Apparatus, Holliston, MA). After 30 min, the occluding thread was removed, and the neck was sutured. For the rat stroke model, young adult male Wistar rats underwent MCAO as previously described [2].

### 2.4 Physiology in Wt and Tg mice

First, anesthesia settings were correlated with arterial blood gases, blood glucose, and blood pressure (normal physiology). Intra-arterial blood pressure was monitored with a CWE, Inc. BPM-832 amplifier (Stoelting Co., Wood Dale, IL); blood gases were determined with ABL 80 Flex (Radiometer America, Inc., Westlake, OH); and blood glucose was measured with Ascensia Breeze glucometer (Bayer Health Care, Toronto, Ontario, CA). Second, cerebral perfusion, arterial blood pressure, blood gases, and blood glucose were monitored during MCAO (ischemic physiology). Measurements were obtained every 15 min; animals were euthanized after 60 min.

### 2.5 Immunolabeling

8μm-thick frozen mouse brain sections at bregma 0.86 were fixed in 4% paraformaldehyde and labeled with anti-MAP2 (ab5392, Abcam, Cambridge, MA) and anti-HuR 3A2 (sc-5261, Santa Cruz Biotechnology, Santa Cruz, CA), 1:1, 000antibodies, per manufacturers’ protocols. Cy3-conjugated (AP194C, Billerica, MA) and Alexa Fluor 488-conjugated secondary antibodies (A11001 and A11008, Life Technologies) were utilized, respectively, per manufacturer's protocols. Rat brain tissue was immunolabeled as described previously [2]. Mouse anti-HuR 3A2 (Santa Cruz) and rabbit anti-KSRP at 1:1, 000 [3] were used with Alexa-Fluor 488 and Cy3-conjugated secondary antibodies, respectively. All sections were counterstained with Hoechst 33258 (Sigma-Aldrich Corp., St. Louis, MO). Fig. 1 shows a schematic of the regions of interest (ROIs) used for the imaging to characterize the tissue responses to ischemia.

### 2.6 Histology

Additional 8μm-thick frozen sections from mouse brains at bregma levels 1.10, 0.86, 0.50, −0.70, −1.22, −1.94, −2.54, and −3.64 were stained with hematoxylin and eosin (HE) and digitally imaged (1.25x objective plus in-line 0.5x video adapter, Olympus).

### 2.7 Western blotting

Brains were removed and dissected. Hemispheres were homogenized; total protein was quantified using the bicinchoninic acid assay (23227 and 23208, Thermo Scientific, Pierce, Rockford, IL). Blots were sequentially probed with antibodies for tubulin 1:50, 000 (ab6046, Abcam) and HuR 1:10, 000 (sc-5261, Santa Cruz). Secondary antibodies, goat anti-rabbit (170-6515) and goat anti-mouse (170-6516) horseradish peroxidase (HRP)-conjugated (Bio-Rad Laboratories, Hercules, CA), were used per manufacturers’ recommendations. Blots were developed using Amersham Enhanced Chemiluminescence Prime (GE Healthcare Bio-Sciences Corp., Piscataway, NJ). Densitometry was performed with Image J 1.46r (National Institutes of Health, Bethesda, MD); bands of interest were standardized to tubulin and quantified as a ratio of ipsilateral to contralateral hemispheres.

## References

[1] Ardelt A.A., McCullough L.D., Korach K.S., Wang M.M., Munzenmaier D.H., Hurn P.D. (2005). Estradiol regulates angiopoietin-1 mRNA expression through estrogen receptor-alpha in a rodent experimental stroke model. Stroke; J. Cereb. Circ..

[2] Carpenter R.S., Iwuchukwu I., Hinkson C.L., Reitz S., Lee W., Kukino A., Zhang A., Pike M.M., Ardelt A.A. (1639). High-dose estrogen treatment at reperfusion reduces lesion volume and accelerates recovery of sensorimotor function after experimental ischemic stroke. Brain Res..

[3] Hall M.P., Huang S., Black D.L. (2004). Differentiation-induced colocalization of the KH-type splicing regulatory protein with polypyrimidine tract binding protein and the c-src pre-mRNA. Mol. Biol. Cell.

[4] Wheeler C., Nabors L.B., Barnum S., Yang X., Hu X., Schoeb T.R., Chen D., Ardelt A.A., King P.H. (2012). Sex hormone-dependent attenuation of EAE in a transgenic mouse with astrocytic expression of the RNA regulator HuR. J. Neuroimmunol..

