# Supplementary material for: Characterization of the transient middle cerebral artery occlusion model of ischemic stroke in a HuR transgenic mouse line
Source: Data Brief. 2017 Oct 17;16:1083–90. doi: 10.1016/j.dib.2017.10.033 (PMC5972843; doi:10.1016/j.dib.2017.10.033)
Supplement: Supplementary file 1 — Supplementary material [file mmc1.docx]

No conflict of interest
